# Test group biases and ethical concerns mar *New England Journal of Medicine* articles promoting HPV screening for cervical cancer in rural India

**DOI:** 10.4103/1742-6413.53466

**Published:** 2009-07-16

**Authors:** R Marshall Austin, Chengquan Zhao

**Affiliations:** Department of Pathology, Magee-Womens Hospital of University of Pittsburgh Medical Center, 300 Halket Street, Pittsburgh, PA 15213, USA

The study conclusions, released from prepublication embargo by the *New England Journal of Medicine (NEJM)* at 5:00 PM on April 1, 2009, were dramatic. “In a low resource setting, a single round of HPV testing was associated with a significant reduction in the numbers of advanced cancers or deaths from cervical cancer.” Furthermore, the study abstract asserted, “No significant reductions in the numbers of associated deaths were observed in the cytology testing group or in the VIA group, as compared with the control group.”[1]

Simultaneously, at 5:00 PM on April 1, 2009, a press release from Qiagen, a Netherlands Holding Company and a manufacturer of the digene HPV test, stated: “Landmark study in New England Journal of Medicine shows HPV testing significantly reduces deaths from cervical cancer, compared to other methods including Pap.”[2] It is now known that the manufacturer received pre-embargo access to contents of the article and the accompanying editorial. The *NEJM* customer service office confirmed on April 14 that pre-embargo release was not from the journal.

A close look at the *NEJM* paper suggests that unexpected biases might have occurred in some of the test group arms of the study which sought to compare a single round of cervical cancer screening with HPV testing, cytology, and visual inspection with acetic acid (VIA) among over 131,746 “healthy women between the ages of 30 and 59 years” in rural India. In fact, the study acknowledged that the positive predictive value (PPV) for detecting CIN 2–3 was 19.3% in the cytologic testing group, higher than 11.3% in the HPV testing group, and study results indicate that essentially the same numbers of cervical cancers were detected after positive screening test results in the cytology arm (88) as in the HPV arm (87). Cervical cancer detection rates were 0.344% in the cytologic testing group and 0.321% in the HPV testing group [Table 1]. Among cervical cancers detected after a positive screening test, more cervical cancers were detected in the earliest and most favorable Stage (IA) in the cytology arm (58/88, 65.9%) than in the HPV arm (45/87, 51.7%), and there were fewer advanced (Stage II+) screening-detected cervical cancers in the cytology arm (10/88, 11.4%) than in the HPV arm (14/87, 16.1%) [Table 1]. The preliminary 2005 publication of study data had earlier reported that cytology had a higher detection rate for CIN 2–3 (1.0%) than for HPV testing (0.7%) and observed the following: “Our present findings indicate that the detection rates of HPV testing did not show any improvement over cytology. Furthermore, the currently available test (HC2) is expensive and requires a relatively sophisticated laboratory operation.”[3] Only the individuals performing HPV testing are specifically described in the 2005 report as having been trained “intensively.” What new information then led to the quite different study conclusions reported in the 2009 publication and the simultaneous manufacturer's press release?

**Table 1:** Cervical cancer cases, cancer stages, and deaths among cancers detected after positive screening tests

*Groups*	*Controls*	*Cytology*	*HPV*
No. of eligible patients	31,488	32,058	34,126
Assigned but not screened	NA	6509	6934
Screened patients	NA	25,549	27,129
Positive screening test detected Ca	NA	88	87
Ca detection rate	NA	0.344%	0.321%
Stage IA	NA	58/88 (65.9%)	45/87 (51.7%)
Stage IB	NA	20/88 (22.7%)	27/87 (28.7%)
Stage II+	NA	10/88 (11.4%)	14/87 (16.1%)
Stage unknown	NA	0/88 (0%)	4/87 (3.4%)
Deaths among positive screening test detected Ca	NA	18/88 (20.5%)	12/87 (13.8%)
Ca detected among controls	118	NA	NA
Ca deaths among controls	64		

The 2009 publication employed the well-recognized “intention-to-treat-principle” in which all eligible patients assigned to a study arm are included in the final analyses for each study arm, whether or not the patients adhere to the research protocol management strategy.[4] Therefore, the final study analyses included in each arm cervical cancer patients who had not been screened, 32 in the HPV arm and 42 in the cytology arm [Table 2]. Since there were 10 more unscreened cancer patients and 5 more cervical cancer deaths in the cytology arm than in the HPV arm and since these unscreened cancer patients predictably had higher rates of cervical cancer, more advanced stages, and less favorable outcomes, the net effect of the inclusion of the larger number of unscreened cervical cancer patients in the cytology arm was to make the cytology arm appear more like the unscreened control arm of the study. Of 54 cervical cancer-related deaths in the cytology cohort, 27 deaths were in the “assigned but not screened” group and another 18 deaths were in patients who had abnormal cytology results. For cervical cancer deaths in the HPV arm, 19% fewer deaths (22) were in the “assigned but not screened” group, and 33% fewer deaths (12) occurred in women with abnormal screening HPV test results. Given the higher PPV and specificity of cytology compared to HPV testing, the outcomes suggest that follow-up colposcopic examinations, tissue sampling procedures, and treatments were not equally effective in the two groups. Significantly inconsistent adherence to management strategies in different study arms may also introduce study bias in investigations, even in studies applying the intention-to-treat-principle.[4]

**Table 2:** Cervical cancer cases, cancer stages, and deaths; cancers detected among assigned but not screened study women

*Groups*	*Controls*	*Cytology*	*HPV*
No. of eligible patients	31,488	32,058	34,126
Assigned but not screened	NA	6509	6934
Ca detected among assigned but not screened women	NA	42	32
Stage IA	NA	0/42 (0 %)	2/32 (6.2%)
Stage IB	NA	5/42 (11.9%)	6/32 (18.8%)
Stage II+	NA	33/42 (78.6%)	20/32 (62.5%)
Stage unknown	NA	4/42 (9.5%)	4/32 (12.5%)
Ca deaths among assigned but not screened women	NA	27/42 (64.3%)	22/32 (68.8%)
Ca detected among controls	118		
Ca deaths among controls	64		

Other aspects of the study data also support the hypothesis that bias was introduced in the study group arms during treatment of patients who developed cervical cancer. For example, the death rate for clinically detected cervical cancer patients in the HPV test-negative group of the HPV arm was 0% as compared to 40.9% among clinically detected cancer patients in the cytology-negative group of the cytology arm [Table 3]. Remarkably, the 0% death rate occurred in the HPV group despite the fact that 62.5% of the HPV test-negative cervical cancers were “advanced stage” (Stage II+). Among patients with positive screening results, similar unexpected outcome differences are also evident [Table 1]. Even though the numbers of cervical cancers in both screen-detected groups were essentially the same (HPV 87; cytology 88), more favorable Stage IA screen-detected cancers were detected in the cytology arm (58) than in the HPV arm (45). Also, fewer unfavorable Stage II+ cancers were screen detected in the cytology arm (10) than in the HPV arm (14). Even after acknowledging the higher negative predictive value of HPV testing reflected in Table 3, deaths in the HPV arm were unexpectedly one-third lower (12) than in the more stage-favorable cytology arm (18). The 2005 publication[3] states that “Women with suspected invasive cancer were referred to the surgical oncology department of NDMCH (Nargis Dutt Memorial Cancer Hospital) for investigations and treatment with surgery and radiation.” The study also notes that “The most common form of treatment for invasive carcinoma in the intervention arm was radical hysterectomy followed by radical radiotherapy.” No data are reported on the extent of similarities or differences in cancer treatments between the test group arms. The effect of the bias of unexpectedly favorable treatment outcomes in the HPV arm contributes to the study conclusion that HPV screening (rather than some other factor) preferentially prevented cervical cancer deaths. The *NEJM* report does acknowledge that “both practitioners and subjects were aware of the study group assignments.”[1]

**Table 3:** Cervical cancer cases, cancer stages, and deaths among cancers detected after negative screening tests

*Groups*	*Controls*	*Cytology*	*HPV*
No. of eligible patients	31,488	32,058	34,126
Assigned but not screened	NA	6509	6934
Ca diagnosed in women with negative screening test results	NA	22	8
Stage IA	NA	2/22 (9.1%)	0/8 (0%)
Stage IB	NA	4/22 (18.2%)	2/8 (25.0%)
Stage II+	NA	15/22 (68.2%)	5/8 (62.5%)
Stage unknown	NA	1/22 (4.5%)	1/8 (12.5%)
Deaths among Ca diagnosed in women with negative screening test results	NA	9/22 (40.9%)	0/8 (0%)
Ca detected among controls	118		
Ca deaths among controls	64		

The *NEJM* publication reports that the study was supported by the Bill and Melinda Gates Foundation, through the Alliance for Cervical Cancer Prevention (ACCP). The paper also states that “no potential conflict of interest was reported.” However, questions have been raised in the peer-reviewed literature as to whether or not the Gates Foundation and the ACCP might have in effect “become enamored with the promise of science and new technologies at the expense of delivering available preventives today”.[5] Questions have also been raised about the partnership between AACP's coordinating organization and the HPV test manufacturer.[5–7] Similarly, the Gates Foundation was recently acknowledged as having funded a large project supporting publication of a special supplemental issue of the journal *Vaccine* entitled “Overview of Human Papillomavirus-Based and Other Novel Options for Cervical Cancer Screening in Developed and Developing Countries.”[8] The HPV test manufacturer is also listed as providing corporate support,[8] acknowledged as “critical for meetings and travel.” The two lead authors of the *NEJM* article and one of the *NEJM* editorial writers are specifically listed among the large number of international contributors. The presence of not even a single cytopathologist among the very large number of contributors supported for international travel and meetings clearly suggests an anticytology bias for the proceedings, one perhaps mirrored in the *NEJM*. The two selected authors of the accompanying *NEJM* editorial[9] also turn out to have co-authored no fewer than 34 PUBMED-cited publications with the former Vice President for Research and Development and Chief Scientific Officer of the HPV test manufacturer. Industry co-authorship was recently cited as the second most common conflict-of-interest in high impact journals such as *NEJM*.[10]

Although the *NEJM* study upon first inspection may be interpreted as applying primarily to low-resource settings where even very limited screening and diagnostic testing are difficult to support, the accompanying editorial,[9] press release[2] and media coverage all strive to suggest otherwise. According to the editorial writers, “The implications of the study in rural India are immediate and global.”[9] According to the *New York Times* (NYT), the study indicates broadly that a “new DNA test outperforms the Pap smear.”[11] Strangely absent from any of the commentary, however, has been any concern that the study included a “control” group of 31,506 “healthy women” who were allowed to largely go without any screening at all during the study, one approved by both local and international “institutional scientific and ethical committees.”[3] Sixty-four women in the “control” group died of cervical cancer along with another 70 unscreened women “assigned to undergo screening,” numbers of cervical cancer deaths substantially in excess of the number which occurred in widely criticized experiments conducted at New Zealand's Auckland National Women's Hospital in the latter half of the last century.[12] The deaths of so many unscreened study “control” subjects made the NYT-quoted comment by a Stanford gynecologist, “The study is another nail in the coffin for Pap smears,” seem particularly insensitive and ironic.[11]

The unexpected increase in the presence of cervical cancer in both the unscreened cytology and VIA arms of the study was a key foundation for the authors' surprising statement that “no significant reductions in the numbers of advanced cancers or deaths were observed in the cytologic-testing group or in the VIA group, as compared with the control group.”[1] When unscreened study cancer victims are included in the “control” group, it becomes clear that significant reductions in advanced stage cervical cancers occurred in virtually all screening group arms [Table 4]. The comparative reductions in advanced stage cervical cancer after a single round of (less expensive) conventional cytology are not significantly different from those achieved with a single round of (more expensive) HPV testing (*P* = 0.2655), even before taking into consideration other study biases. Furthermore, the study's cytology screeners were trained for only 3 months, one-fourth of the usual year-long training for cytotechnologists. This was almost certainly suboptimal to confront the challenging conditions in the rural Osmanabad district. Pap screening, when successfully implemented, reduces cervical cancer rates by 60–90% within 3 years of introduction to populations that have not been screened; these reductions in incidence and mortality are consistent and dramatic across populations.[5,13] Even a single round of screening with conventional cytology and limited training in rural India appears to have been remarkably effective, despite the published interpretations of study data in the *NEJM*. As noted by many of the same authors in their 2005 publication, “Our results clearly show that good quality cytology can be implemented even in a rural setting of a developing country with reasonable investment, while HPV testing does not give any better detection of CIN2/3 lesions, despite the higher investments.”[3] Careful reading of the 2009 publication data and assessments of the numerous internal biases suggest that the widely reported conclusions may be significantly misleading.

**Table 4:** Advanced stage (II+) cervical cancers: Unscreened control group and unscreened assigned study group vs. screened study group women

*Groups*	*Controls and assigned but not screened*	*VIA*	*Cytology*	*HPV*
No. of eligible patients	31,488	34,074	32,058	34,126
Assigned but not screened	20,752 (all arms total)	7309	6509	6934
Stage II+ Ca after positive screening test	NA	35	10	14
Stage II+ Ca after negative screening test	NA	19	15	5
Total screened stage II+ Ca	NA	54	25	19
Stage II+ Ca after being assigned but not screened	85	NA	NA	NA
Stage II+ Ca “controls”	82	NA	NA	NA
Total Stage II+ Ca controls and assigned but not screened	167	NA	NA	NA

## COMPETING INTEREST STATEMENT MADE BY ALL AUTHORS

No competing interest to declare by any of the authors.

## AUTHORSHIP STATEMENT BY ALL AUTHORS

All authors of this article declare that we qualify for authorship as defined by ICMJE http://www.icmje.org/#author.

Each author has participated sufficiently in the work and take public responsibility for appropriate portions of the content of this article.

Each author acknowledges that this final version was read and approved.

## EDITORIAL / PEER-REVIEW STATEMENT

To ensure integrity and highest quality of CytoJournal publications, the review process of this manuscript was conducted under a double blind model [author(s) are blinded for reviewer(s) and reviewer(s) are/is blinded for author(s)] through automatic online system. CytoJournal is an open access platform to publish any peer-reviewed scientific point of view in Cytopathology and related areas. The views reflected in the article are those of authors alone and does reflect any agreement or disagreement by the journal and its publisher.
